# Planning, implementation and operation of a personalized patient management system for subjects with first suspect of cancer (OnkoNetwork): system description based on a qualitative study

**DOI:** 10.1186/s12913-019-3957-9

**Published:** 2019-02-21

**Authors:** János G. Pitter, Marcell Csanádi, Annamária Szigeti, Gábor Lukács, Árpád Kovács, Mariann Moizs, Imre Repa, Antal Zemplényi, Thomas Czypionka, Markus Kraus, Maureen P. M. H. Rutten-van Mölken, Zoltán Kaló

**Affiliations:** 1 Syreon Research Institute, Budapest, Hungary; 2 OnkoNetwork Office, Móritz Kaposi General Hospital, Kaposvár, Hungary; 3 Department of Oncology, Móritz Kaposi General Hospital, Kaposvár, Hungary; 4 Doctoral School, Faculty of Health Sciences, University of Pécs, Pécs, Hungary; 5 Móritz Kaposi General Hospital, Kaposvár, Hungary; 6 Healthcare Financial Management Department, University of Pécs, Pécs, Hungary; 7 Institute for Advanced Studies (IHS), Vienna, Austria; 8 Erasmus School of Health Policy and Management, Institute for Medical Technology Assessment, Erasmus University Rotterdam, Rotterdam, the Netherlands; 9 Department of Health Policy and Health Economics, Eötvös Loránd University, Budapest, Hungary

**Keywords:** Integrated care, Patient pathway, Cancer, Diagnostics, Delay, OnkoNetwork, SELFIE, Qualitative study, Thick description

## Abstract

**Background:**

OnkoNetwork is a recently established integrated care model with a personalized pathway system to manage patients with first suspect of a solid tumour in secondary care, that evolved as a regional initiative in Hungary. The primary aim of OnkoNetwork is the improvement of clinical outcomes via timely access to quality assured and defragmented healthcare services. The Horizon 2020 funded SELFIE project has selected OnkoNetwork for in-depth qualitative and quantitative evaluation. The aim of this study was to provide a qualitative evaluation of OnkoNetwork along the six components of the SELFIE conceptual framework: 1) service delivery, 2) leadership and governance, 3) workforce, 4) financing, 5) technologies and medical products, and 6) information and research.

**Methods:**

Analysis of published and grey programme documentation, followed by 20 semi-structured interviews with representatives of programme initiators, general and financial managers, involved physicians and non-physician professionals, patients and their informal caregivers. Transcripts of all interviews were analysed by Mayring’s content analysis method by two independent researchers.

**Results:**

This study yielded the first comprehensive description of the programme. OnkoNetwork is a blue dahila in Central and Eastern Europe, providing timely and quality-assured healthcare services for the target patients by personalized patient path monitoring and management in a financially sustainable manner without macro-level financing of its operation. Innovative professional roles were implemented for non-physicians and physicians, and a supporting information technology application was developed.

**Conclusions:**

This paper provides a systematic description of OnkoNetwork on the six components of the SELFIE conceptual framework for integrated care in multimorbidity to understand how and why OnkoNetwork was implemented and cares (better) for its patients. Because integrated care models are designed and adjusted to their specific local needs and context, those few successful and sustainable models that were established in Central and Eastern European countries represent important benchmarks for other initiatives in this region. Experience with OnkoNetwork during its planning, implementation and operation including the description of key success factors and barriers as perceived by various stakeholder groups, may support the development of further integrated care models especially in countries with similar economic status and healthcare settings.

**Electronic supplementary material:**

The online version of this article (10.1186/s12913-019-3957-9) contains supplementary material, which is available to authorized users.

## Background

Prevalence of multi-morbidity is rapidly increasing in Western populations. As opposed to fragmented and single disease focused health care provision, the need for personalized integrated care is more and more recognized [1–3]. A recently published new framework on integrated care for multi-morbidity organised elements of integrated care that had previously been reported to contribute to its effectiveness into the six WHO components of health systems, thus providing a model for a system-level approach [1]. The establishment of this framework was an important initial achievement of the Horizon2020 funded SELFIE project (Table 1 near here). This conceptual framework guided the detailed qualitative description and the currently ongoing quantitative evaluation of 17 selected integrated chronic care models for persons with multi-morbidity, in 8 European Union Member States participating in SELFIE, including Austria, Croatia, Germany, Hungary, the Netherlands (coordinator of SELFIE), Norway, Spain, and the UK. A brief description of the challenges faced by the SELFIE project and four other EU funded projects on integrated care for multimorbid patients (SUSTAIN, ACT@Scale, SCIROCCO, JA-CHRODIS/CHRODIS-PLUS) was given by Rutten-van Mölken et al. [2]. This paper focuses on one of the Hungarian integrated care programmes analysed in SELFIE, named OnkoNetwork.

**Table 1:** About the SELFIE project

SELFIE (Sustainable int E grated chronic care mode L s for multi-morbidity: delivery, FI nancing, and performanc E) is a Horizon2020 funded EU project that aims to contribute to the improvement of person-centred care for persons with multi-morbidity by proposing evidence-based, economically sustainable, integrated care programmes that stimulate cooperation across health and social care and are supported by appropriate financing and payment schemes.	
More specifically, SELFIE aims to:	
• Develop a taxonomy of promising integrated care programmes for persons with multi-morbidity	
• Provide evidence-based advice on matching financing/payment schemes with adequate incentives to implement integrated care	
• Provide empirical evidence of the impact of promising integrated care on a wide range of outcomes using Multi-Criteria Decision Analysis	
• Develop implementation and change strategies tailored to different care settings and contexts in Europe, especially Central and Eastern Europe	
The SELFIE consortium includes eight organisations in the following countries: the Netherlands (coordinator), Austria, Croatia, Germany, Hungary, Norway, Spain, and the UK. www.selfie2020.eu [Grant Agreement No 634288]. Accessed 20 Feb 2019.	

OnkoNetwork is a recently established integrated care model with a personalized care pathway to manage patients with first suspect of a solid tumour, that evolved as a regional initiative to tackle severe coordination deficits within the healthcare system in Hungary. OnkoNetwork aims to improve clinical outcomes via timely access to quality assured and defragmented healthcare provision. This model was established in 2014 by the Móritz Kaposi General Hospital in Somogy county, Hungary. To support the documentation and management of individualised care pathways, a tailor-made IT system was developed, and new professional roles were established for both non-physicians and physicians [4, 5]. Target patient groups in OnkoNetwork have high rates of clinically relevant comorbidities, which explains why the programme was selected by the SELFIE consortium as one of the most promising integrated care models for patients with multi-morbidity in Hungary. OnkoNetwork focuses on improving timely access to diagnosis and start of cancer treatment because this is associated with improved patient survival in many types of cancer e.g. in lung cancer [6, 7], oral squamous cell cancer [8], head and neck squamous cell carcinoma [9, 10], and breast cancer [11].

The aim of this paper is to give a systematic description of OnkoNetwork on the six components of the SELFIE conceptual framework for integrated care in multi-morbidity: 1) service delivery, 2) leadership and governance, 3) workforce, 4) financing, 5) technologies and medical products, and 6) information and research. The full report of the qualitative study is available from the SELFIE website (http://www.selfie2020.eu) as a thick description [12]. This study can support the creation and implementation of future individual care pathways, especially in countries with similar healthcare systems and economic status to Hungary.

## Methods

### Thick description

Thick description is a qualitative empirical approach introduced by Gilbert Ryle [13] and exploited in the 1970s by Clifford Geertz as a qualitative method to investigate implicit social practices [14]. Geertz’ methodological and conceptual work influenced empirical research in several disciplines [15], and thick description became a well-established approach in sociology including care practice research [16]. In brief, first the “hard facts” about the investigated phenomenon are described, then it is supplemented with “soft facts” answering the “how” and “why” questions and allowing a deeper understanding of the operational practice. In the SELFIE project, “hard” and “soft” facts were collected both from document analysis and from stakeholder interviews.

### Document analysis

The analysed documents included Hungarian language publications on OnkoNetwork as identified via internet searches in PubMed and Google, and internal documents provided by the managers of OnkoNetwork (official documents of the programme and written correspondence). Relevant data were extracted, including but not limited to the scope and organisational structure of the programme (e.g. official name, implementation milestones, aims, target patients, service delivery, involved disciplines and professions, organizational form, and IT support tools).

### Selection of interview participants

Altogether 20 interviews have been conducted with representatives of the key stakeholder groups of OnkoNetwork: one programme manager, two programme initiators (opinion leaders that participated in initiating, conceptualising and planning the programme), one financial expert of programme funding, four physicians, eight non-physician health care professionals of OnkoNetwork, two patients, and two informal caregivers (family members of patients). Since OnkoNetwork is without specific macro-level financial incentives or regulatory or policy support system, representatives of the National Health Insurance Fund and State Secretariat for Health at the Ministry of Human Capacities were not considered as relevant stakeholders and were not interviewed about their views on OnkoNetwork. Selection of and proportions across these stakeholder groups were guided and approved by the corresponding work package leaders of the SELFIE consortium, in a purposive rather than convenience-oriented manner; selection of all interviewers preceded the start of analysis, therefore deciding on stakeholder group proportions was not influenced by saturation of information across interviews. From the corresponding stakeholder groups, the interviewees themselves were selected and invited by the OnkoNetwork management team, based on their personal involvement in OnkoNetwork related activities. For the patient and informal caregiver stakeholder groups, multimorbidity was also a selection criterion. The number of interviewed physicians and non-physicians was slightly higher than initially planned (3 and 5, respectively), due to a few snowball participants. None of the invited interview participants refused to take part in this study.

### Interviews

The interviews were conducted by two male employees of Syreon Research Institute, the SELFIE research team in Hungary (authors JGP and MCs), with MD, PhD and with MSc credentials, respectively. All interviews were conducted face-to-face without making field notes, with voice recording, in Hungarian language. The 30- to 90-min interviews (median 60 min) took place in the facilities of the Móritz Kaposi General Hospital at Kaposvár, in July 2016, in a quiet, light and door-separated room without through traffic and equipped with a table and chairs. Beyond the interviewee and the two interviewers, no one else was present in the room during the interviews, except for one interview where two family members of the same patient arrived and preferred to be interviewed together. The interviews were semi-structured. Wording of the questions was guided by a written interview protocol that determined the structure of the interviews, and defined stakeholder group specific themes according to the SELFIE conceptual framework (Table 2; the Interview Protocol is provided in the Additional file 1). The interview protocols were standardized across the SELFIE consortium but were flexible enough to further discuss important topics that came up during the interviews, due to the semi-structured approach. The corresponding Work Package leader provided personal training and detailed guidance how to get prepared for the interviews. Pilot interviews hence were not deemed necessary. In general, all interview protocols were structured as follows: first a brief introduction about SELFIE and objectives of the interview were presented, followed by signing the informed consent for recording (all invited interviewees agreed to do so). The first research questions dealt with the participant’s qualification and his/her role in OnkoNetwork. Next questions covered specific topics of each stakeholder group (Table 2). The last question was about the interviewee’s perception on the most important achievement of and the most important future challenge for OnkoNetwork [12].

**Table 2:** Key focus areas of the interviews by stakeholder groups

Stakeholder groups	Key focus areas of the interviews
Programme Manager	Personal contribution to the programme; Programme achievements in patient-centred care
Programme Initiators	Basic idea and the conceptual framework of the programme
Financial experts / payers	Considerations about the financing decision; Experiences with the financing and payment systems
Physicians	Changes in physicians’ work practice due to the programme
Non-physician professionals	Personal description of last workday; Experience with active participation of patients
Informal caregivers	Personal description of care work; Personal involvement in the programme
Patients	Personal description of care experience; Personal experience in self-management

Interview protocols were not shared with the interviewees. All participants were interviewed only once.

### Interview analysis

Based on the audio recordings, interviews were transcribed and analysed by Mayring’s content analysis method [17], using the following steps of abductive interpretation: selection of units for analysis; paraphrasing; making short forms; and creating categories around common themes when possible.

Short forms and category constructs were developed by the two researchers who conducted the interviews, in English language, in Microsoft Excel. Their findings were pooled to reduce the risk of “blind spots” of a single analyst in the abstraction process. All recognised constructs were assigned to one or more of the eight domains (prespecified by the SELFIE consortium) covering different aspects of the evaluated programme, such as (i) implementation aims and history, (ii) delivery of care; (iii) relationships with other care providers; (iv) role of information and communication technology (ICT) applications; (v) self-management interventions; (vi) new professional roles; (vii) existing evidence on impact; and (viii) experience with financing / payment schemes. Each paraphrase could be analysed in multiple contexts, depending on the complexity of its content. Because these domains covered the six components of the SELFIE framework, the information gathered in each domain was later assigned to one of SELFIE framework components, except for implementation history that corresponds to the framework as a whole. Finally, an integrated description of the system components was derived from the document and interview analysis, explicitly contrasting the views of various stakeholder groups when divergence was observed. Findings of the interview analysis were presented to those interview participants who were co-authors of the Thick Description and this manuscript; all of them reviewed and approved the report findings without substantial changes.

### Quality assurance

To ensure the proper implementation of the selected methodology in the description of all the 17 selected models, the SELFIE work package leader provided other SELFIE partners with detailed methodology guidance in three waves. In the preparatory phase, written methodological guidance materials were distributed to all partners. Then a training course was offered to interviewers and researchers directly involved in the thick description process, before conducting the interviews. Finally, the reports of the 17 thick descriptions were reviewed by the work package (co)leaders to ensure that their results were clearly presented and to harmonise their structure. Reporting quality in the present paper was cross-checked to comply with the consolidated criteria for reporting qualitative studies using the 32-item COREQ checklist [18].

## Results

Findings of the thick description of OnkoNetwork are described along the six components of the SELFIE framework (i.e. service delivery, leadership and governance, workforce, technologies and medical products, information and research, and financing), preceded by a short description of its planning and implementation history.

### Implementation of OnkoNetwork

#### Planning phase

**Fig. 1:**
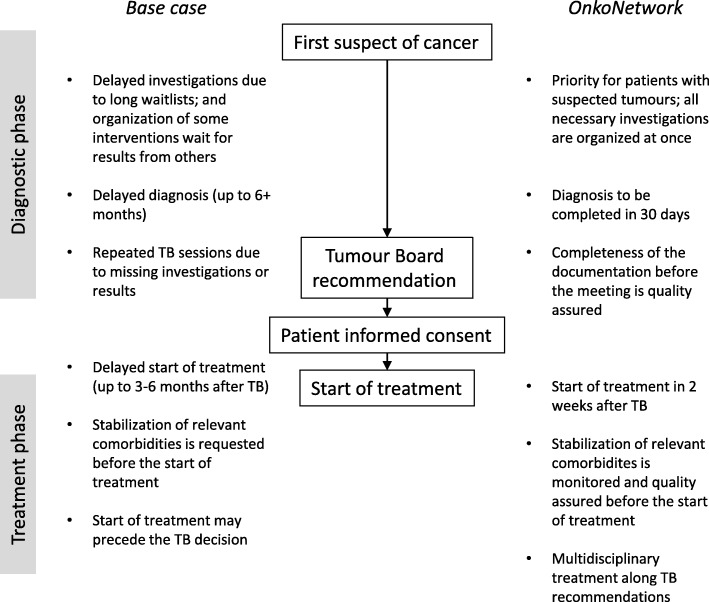
Integration of healthcare service providers in OnkoNetwork. Modified from [4]

The idea of OnkoNetwork first came up in July 2014 when the Strategic Director of the Móritz Kaposi General Hospital set up a small interdisciplinary team to understand the root causes of the unexpectedly high cancer mortality in Hungary [19]. This county hospital is responsible for the comprehensive oncology care of about 500,000 inhabitants in Hungary, in close cooperation with the Kaposvár University Health Center (responsible for CT, MR, PET CT, PET MR diagnostics and also for radiotherapy), with outpatient cancer care departments of the Hospital in other locations (Siófok, Nagyatád, Marcali), and with a radiotherapy unit in Mosdós [4]. The team looked for quantitative indicators with high variability that could be controlled by the above institutions and identified two parameters calling for improvement. First, diagnostic delay in the centre (time from the first presented suspect of cancer in the medical system of the participating institutions to the Tumour Board meeting with final diagnosis) was found to be as long as 6 months in many cases. Secondly, the treatment initiation delay (time from final diagnosis to the first treatment day) was also longer than the optimal time window for many patients. To reduce these time windows in the catchment area of the Hospital, a new system to manage and optimise patient pathways was proposed as a local initiative. The planning phase was based on intensive teamwork involving both the hospital management and the heads of all hospital departments contributing to cancer diagnosis or treatment (listed on Fig. 1). After repeated meetings and strategic discussions, the ultimate objective of OnkoNetwork was framed as *“provision of timely and equitable access to comprehensive and integrated oncology care”*. Potential economic benefits (such as a reduction in average treatment costs) were not the primary focus in the planning phase. OnkoNetwork was based on several principles. First of all, quality assurance was facilitated by the development and implementation of organ-specific, evidence based clinical protocols. Secondly, tracking of each patient by an identified responsible physician had to be made continuously to prevent patients from being lost in the system. Thirdly, quantitative indicators were defined: the target for a conclusive Tumour Board decision on the proposed therapy was set at a maximum of 30 days of enrolment into OnkoNetwork, and thereafter the treatment should be started within 14 days. To support these principles, development of a new tailor-made IT system was decided for real-time collection and monitoring of the clinical documentation of oncology patients [4]. Given the volume and complexity of the existing medical IT system of the Hospital, the realistic ambition was to develop the new IT tool running in parallel with pre-existing medical IT applications, rather than as a replacement of them.

#### Implementation history

OnkoNetwork was implemented in a pre-planned change management process. The first implementation step was the development of organ-specific diagnostic algorithms: available clinical protocols that typically contained a couple of optional investigations to be selected along not fully explicit criteria were translated into organ-specific diagnostic algorithms with standardized lists of all necessary investigations by the organs affected. Harmonized process descriptions of hospital departments were also developed. After consensus was achieved in these aspects, the development of a customized IT application (OncoLogistic) was started, in parallel with the thorough reorganization of teams and harmonization of oncology care in all relevant departments [4]. Since no relevant prior experience could be identified in Hungary, the workflow description and the definition of new professional roles were started from scratch, based on thorough discussions involving all stakeholders. An OnkoNetwork Office (see below) was established and OncoLogistic functions were continuously revised and fine-tuned. OnkoNetwork was officially launched in November 2015, after a 1-month test period.

After this brief overview of the planning and implementation phases, the next sections describe the OnkoNetwork model along each component of the SELFIE framework.

### Service delivery

#### Target population

The target population of OnkoNetwork consists of adult (≥18 years) patients with new suspect or new diagnosis of solid tumours (an ICD disease code starting with “C” or “D”, except for haematology malignancies, and some rare tumours that are referred to specialized national centres) in the catchment area of the Kaposi Mór General Hospital at Kaposvár. This catchment area roughly overlaps with Somogy county of Hungary with a 500,000 population, including patients visiting the Oncology outpatient units of Municipal Hospitals at Nagyatád and Siófok. Having chronic comorbidities is not an enrolment criterion in OnkoNetwork, the most frequent chronic comorbidities in the enrolled population are cardiovascular diseases, hypertension, and diabetes occurring in about 49, 26, and 11%, respectively. Diagnosis and adequate treatment of comorbidities interfering with cancer care are inevitable in many cancer patients, e.g. pacemakers need to be switched off or removed during high frequency ablation; patients receiving cardiotoxic chemotherapy require cardiologic control; or metformin treatment of diabetic patients need to be discontinued before CT scan to avoid kidney damage, as a few examples.

#### The care processes

##### Patient enrolment

OnkoNetwork offers priority in care, personalized care pathways, and timely access to quality assured healthcare services for enrolling patients upon their first appearance in the hospital’s medical system. At the entry visit, the OnkoNetwork care model is explained to the patient and the enrolment is offered upon signing an informed consent form. Most of the invited patients has decided to join OnkoNetwork so far.

##### Personalized diagnostic plans

After filling in a standardized questionnaire on anamnesis, risk factors, comorbidities, and current medications, a responsible physician is appointed to each enrolled patient. The diagnostic plan is guided by organ- and disease-specific protocols, and the responsible physician can deviate from the diagnostic protocols only with specified and documented reasons. Diagnostics and management of comorbidities must also comply with timelines established for cancer diagnostics and start of cancer therapy.

##### Coordination and documentation of diagnostic visits

Before the OnkoNetwork era, arrangement of diagnostic visits was a huge burden for physicians with inefficient phone calls and fighting for earlier dates. By the introduction of OnkoNetwork and the declaration of priority status for cancer patients, early dates are continuously available and can be booked through a web application or phone by non-physician staff members. The reduced diagnostic time windows for OnkoNetwork patients were not reported to have negative influence on waiting times of other patients. Preferences of patients are taken into account, e.g. when scheduling multiple diagnostic visits on the same day to decrease unnecessary travel costs and time. Documents for referral to further diagnostic tests are given to patients during their first visit, in order to avoid repeated hospital visits merely to organize the forthcoming diagnostic procedures.

##### Tumour board meeting

The Tumour Board (also called “Onkoteam” in Hungary) is a multidisciplinary board of the Hospital consisting at least of a clinical oncologist, a surgeon and a non-surgeon specialist of the affected organ system, a (molecular) pathologist, a radiologist, a radiotherapist, the patient’s treating physician, and a coordinator of the team. The composition of this board is defined by the law with the primary responsibility of providing multidisciplinary personalized diagnostics and treatment plans for cancer patients. Once all necessary diagnostics are completed the case has to be referred to the next Tumour Board meeting, within 30 days of entering OnkoNetwork [4, 5]. OnkoNetwork did not change the composition or frequency of Tumour Board meetings but smoothed its operation by validating the completeness of patient documentation prior to the meeting and providing online access to diagnostic results at the meeting. Participation of patients in the Tumour Board meetings is exceptional, as it would not be technically feasible due to time pressure on Tumour Board members. In a few days after the Tumour Board meeting, the responsible physician informs patients about their treatment plans. In many cases, alternative treatment options are discussed with the patient who may accept, reject or occasionally adapt the proposed treatment strategy.

##### Treatment and follow-up

In OnkoNetwork, the agreed cancer therapy must be initiated within 14 days after the conclusive Tumour Board meeting. Any delay in referring a case to the Tumour Board, or in therapy initiation must be justified by the responsible physician. To ensure that patients are treated as agreed in the multidisciplinary Tumour Board meeting, it is not allowed to start any cancer therapy prior to the decision of the Tumour Board. Nevertheless, clinical management of relevant comorbidities does not have to wait for the Tumour Board decision. After the completion of acute interventions, a follow-up Tumour Board meeting is scheduled, and follow-up plans are made with regular control diagnostics. Administrators at the OnkoNetwork Office trace those patients who do not appear at these control visits or who have delayed Tumour Board meetings.

### Leadership and governance

**Fig. 2:**
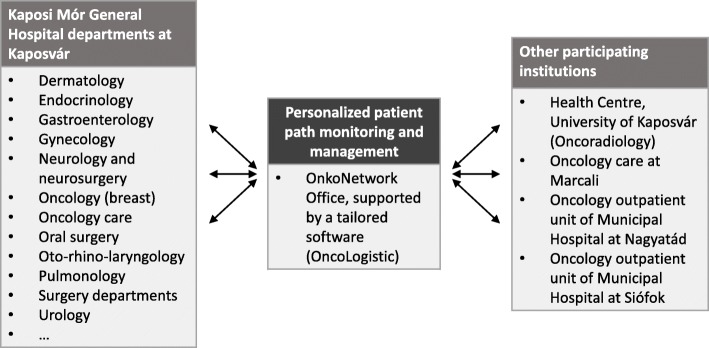
Core features of OnkoNetwork, as opposed to the routine practice in Hungary. TB, Tumour Board

The management of the Móritz Kaposi General Hospital played a proactive and leading role in the conceptual development, planning, and implementation of OnkoNetwork. In the implementation phase the Hospital General Director delegated the OnkoNetwork upper management responsibility to the co-initiator Strategic Director of the Hospital, who also acts as a professor at the Kaposvár University Health Centre [4]. Note that most OnkoNetwork activities are hosted by the Móritz Kaposi General Hospital, which – as a county hospital – is equipped to fulfil a leadership role in organizing health care in the region and has a relatively centralized governance structure, as opposed to medical centres of universities with greater independence of various clinics and distinguished professors. The leadership team made continuous efforts to achieve decisions by consensus at interactive meetings, and any feedback was seriously considered in the implementation process. Program launch was approved by the Board of Directors, and was announced by the General Director of the Hospital [4, 5]. The organizational structure of OnkoNetwork, and its core features are depicted in Figs. 1 and 2.

Fear of change and protection of status quo based on individual or departmental incentives were noticeable upon implementation. At initiation, many physicians had to learn that they could not make decisions alone anymore, and they had to work with other health care professionals as a team in order to provide integrated multidisciplinary care. The upper management successfully overcame the initial resistance by continuous reinforcement of OnkoNetwork’s principles. OnkoNetwork was implemented according to a change management process with systematic amendments of work descriptions. Education and training, written instructions to all professionals, and learning by doing opportunities in the test period also contributed to the successful implementation. The availability of all necessary diagnostic and treatment modalities for a complex oncology care centre, as well as the underlying infrastructural developments and the consistent development strategy of the hospital to provide cutting edge oncology care in the last 10–15 years were also strong success factors for OnkoNetwork at Kaposvár.

Monitoring of OnkoNetwork operations is accomplished at multiple hierarchical levels, ranging from a dedicated project office (OnkoNetwork Office) consisting of 4 full-time administrators and an Office Manager, through two supervisor physicians, to the top management. The Strategic Director does not have to be involved in OnkoNetwork operation on a daily basis but is informed every week about all patients with delays. His unique leadership position and strong commitment to OnkoNetwork are important facilitators for implementation, permanent operation and future development of the model. The Hospital General Director may also intervene in the rare escalated cases, e.g. when noncompliance is identified at a department level. Macro-level approval or adjustment of the external legal framework was not necessary, due to hospital autonomy.

Political support to OnkoNetwork by the central government was not visible for a long time. The programme was first recognized by the Hungarian Health Economics Association, which has been playing an important role in the promotion of evidence based health policy [20]. Subsequently, the general media and patients with access problems in other regions acknowledged the potential benefits of the programme. The opinion of other oncology centres on OnkoNetwork in the region was initially negative, but later they understood that it does not aim to compete for their patients. Finally, the importance and benefits of OnkoNetwork were recognized by the State Secretariat at the Ministry of Human Capacities. The OnkoNetwork team received an award from the Minister of Human Capacities for their exemplary professional work in healthcare in 2016. Scaling up OnkoNetwork to other regional centres (i.e. county hospitals with complex oncology care) is on the political agenda in Hungary.

### Workforce

OnkoNetwork introduced new professional roles and reshaped existing ones as overviewed below.

#### Changes in the roles of department physicians

Before OnkoNetwork, the organization of diagnostics was a time consuming and stressful administrative burden on physicians who had to fight for early visit dates for their patients. OnkoNetwork gave priority status for patients with suspected cancer throughout the Centre and passed this organization task to selected department administrators. Department physicians allocate their spared hours to more extensive patient communication, to the treatment of more patients in a day, or to other professional tasks. On the other hand, the programme limited the independence of physicians and forced them to collaborate with each other along personalized multidisciplinary diagnostic and treatment plans. To ensure the continuity of care, all enrolled patients have a named responsible physician who is competent to decide on their diagnostic tests and is responsible for any delay in diagnosis and therapy initiation. The responsible physician changes over time in the patient path: is typically a physician from the relevant organ-specific department in the diagnostic phase, a surgeon in the perioperative stage, and an oncologist during chemo- and radiotherapy episodes. The responsible physician must be available for discussions with supervisor physicians, and is also available for departmental OnkoNetwork administrators, but shall not be contacted by OnkoNetwork Office administrators (see the description of these new roles below).

#### Changes in the roles of departmental non-physician staff members

One to three assistants or administrators have been selected, appointed and trained in the relevant departments of the Hospital to become departmental OnkoNetwork administrators. Their related tasks are 1) case finding, i.e. to invite and enrol candidate patients from the target population; 2) to fill in the OnkoNetwork questionnaire at programme entry based on a patient’s interview; 3) to schedule visits for protocol driven and responsible physician initiated diagnostic tests; 4) to record data into OncoLogistic, including the responsible physician’s explanation for any delay or deviation from the protocol; and 5) to monitor therapy initiation after the Tumour Board decision. Most OnkoNetwork administrators in the departments received their new roles on top of existing ones, except for departments with highest enrolment rates where the OnkoNetwork administrator function can be a full-time position.

#### A new non-physician role: OnkoNetwork office administrators

In contrast to departmental OnkoNetwork administrators described in the previous section, OnkoNetwork Office administrators do not belong to any hospital department. Interestingly, most OnkoNetwork Office Administrators did not have prior education or experience in healthcare service provision but were hired based on their good organization and communication skills. Their main role is monitoring and managing patient pathways directly and through contacts with OnkoNetwork administrators in linked departments, as supported by the tailored IT application OncoLogistic. In a typical day, OnkoNetwork Office administrators identify about 40–50 OnkoNetwork candidate patients based on their recorded ICD codes, and systematically check whether they are invited and enrolled into OnkoNetwork by the departmental OnkoNetwork administrators. They also look after all enrolled patients with a delay in their pathway on a daily basis. When they identify a patient with an apparent delay, they first clarify whether it is due to missing data transfer across medical IT systems. If not, they turn to the OnkoNetwork administrator(s) of the relevant department for clarification. They always accept the received justification but secure that it is recorded in OncoLogistic. OnkoNetwork Office administrators notify the supervisor physicians (see below) about any unsolved issue.

#### A new physician role: Supervisor physicians

One supervisor physician is working at the Hospital and another one at the Kaposvár University Health Centre. Their primary role is to negotiate those OnkoNetwork related issues with department physicians that could not solved at the non-physician level. Any remaining unsolved cases are referred to the Strategic Director of the Hospital on a weekly basis. The supervisor physicians do not interfere with medical decisions of department physicians, but request justification for unexplained delays in diagnostics and care. The selected supervisors have a calm and helpful personality that efficiently relieves the psychologic stress on the involved non-physician team members.

#### Barriers and success factors for the new roles

Since no relevant prior experience could be identified in Hungary, the workflow description and the definition of new professional roles were started from scratch. Involvement of all stakeholders in the planning, and building consensus agreements were important success factors in the planning phase. Training for the new roles included interactive plenary lectures and practice oriented technical presentations on model objectives and workflows. Written guidance to the custom IT tool OncoLogistic was also distributed, but its technical language could not be easily understood by several staff members. However, a few months of learning by doing experience was sufficient for all administrators to get fluent in OncoLogistic.

At implementation, some surgeons were irritated as they could not make independent decisions on the treatment (e.g. when to take a histology sample). Their resistance was overcome by the involvement of department heads in consensus agreements, strong commitment of the management, and early perceived benefits of the model, e.g. scheduling the diagnostic visits by OnkoNetwork administrators. A further barrier came from the culture of many physicians who were not prepared for the new professional roles of non-physician colleagues. OnkoNetwork did not change this attitude of physicians but circumvented this situation by the establishment of the supervisor physician role in the model. Supervisor physicians are much more accepted than non-physician team members as partners by physicians.

#### Relationships with other providers

In Hungary, patients with more complex medical needs have long waitlists to their definitive care even within a single institution [4, 5]. Accordingly, key opinion leaders of OnkoNetwork decided to focus first on the integration of care processes that were under their control in the region, i.e. hospital care and secondary outpatient care provision. Nevertheless, they recognized that integrated care should cover the holistic needs of patients including prevention, primary healthcare, social care, psychological support, physiotherapy and dietary advice. The OnkoNetwork Management identified primary care and cancer screening programmes as key contributors to early detection of suspected cancer. Note that cancer screening programmes in Hungary are organized by special health promotion offices, without data linkage to oncology centres and without follow-up system of the identified positive screening cases. Regional integration of OnkoNetwork with cancer screening programmes is an important ambition of the OnkoNetwork management.

### Technologies and medical products

Before OnkoNetwork, a range of ICT applications had already been implemented in the hosting institutions. The goal of OnkoNetwork was not to replace these IT systems, but to develop a new tailor-made IT application (OncoLogistic) in parallel with these systems, to support documentation and management of patient care pathways, and performance monitoring of OnkoNetwork. Every OncoLogistic user has a personal ID to log in, and all data entries can be traced back to users. Standardized, organ-specific diagnostic algorithms are integrated into OncoLogistic so that the scheduling of diagnostics could be passed to non-physician users using this platform. Patients have no access to OncoLogistic at present.

The biggest challenge in the IT development was to achieve the connectivity of OncoLogistic to existing medical IT systems. The key issue with data transfer across IT systems was the lack of appropriate medical IT standards. On one hand, diagnosis-related-groups and intervention codes had been created only for financial purposes in Hungary and are not sufficiently graded to differentiate between disease and intervention subtypes in patient pathway algorithms. On the other hand, independent IT software developments resulted in a multitude of medical IT applications that are in use at different providers but are not compatible with each other.

Differences in professional languages, terms and ways of thinking occasionally also resulted in barriers when the development of diagnostic algorithms was approached by clinicians and IT engineers. A mediator with thorough experience in both disciplines, and the openness of upper management to IT perspectives could facilitate resolution in such situations.

Development of OncoLogistic was a big technical challenge as shown above, however, this tailor-made IT application after its launch became an important success factor in the implementation of OnkoNetwork, empowering the relevant stakeholders with up-to-date reports, and organizing their daily work on a well-documented and transparent platform.

Departmental OnkoNetwork administrators entered data both into OncoLogistic and into the hospital’s medical system in parallel, due to the limitations of the established interface between these systems (a pilot update version of OnkoNetwork with improved connectivity was under development). Data export / import in OncoLogistic was under continuous fine-tuning. Another development direction for OncoLogistic was the integration of treatment algorithms. Future development opportunities for OncoLogistic include building interfaces to all healthcare IT systems in Hungary, and its regional scale-up in Hungary and beyond. This tool was developed in a way that allows its rapid adaptation to other regions / healthcare systems, and it is multilingual, theoretically capable to import data from any system.

### Information and research

No formal evaluation reports on OnkoNetwork are available so far. The management expects that at least a few years of follow-up will be necessary to evaluate its clinical and economic impacts. Moreover, benefits of the programme are expected to be boosted once the cancer screening system and primary care will be connected, shortening the patient delay before first secondary care visit. Dimensions of the expected impact of OnkoNetwork are summarized below, along with the currently available very limited data on timeliness of care.

#### Clinical outcomes

Initial analyses suggest shortened timelines for diagnosis and treatment initiation, as expected. The mean time from initial indicative diagnosis in the hospital to Tumour Board meeting is reported to be 21–22 days in OnkoNetwork with a standard deviation of 14 days. Before OnkoNetwork, the mean duration was 64 days with a standard deviation of 40 days in the Hospital. For comparison, the mean duration of cancer diagnosis in other Hungarian hospitals can be as high as 110 days [12].The interviewees expected that OnkoNetwork will result in better patient compliance in the diagnostic phase, less incentives for patients to visit private healthcare providers in order to speed up the diagnostic process, faster diagnosis and treatment of comorbidities, lower frequency of inconclusive Tumour Board meetings, earlier start of treatment, lower proportion of patients with cancer metastases at diagnosis, longer patient survival, and/or better nutritional status of patients with head-neck tumours during therapy. Some interview participants suggested that more patients would receive surgical care due to a shift in cancer stage distribution at diagnosis towards less progressed cases, while the opposite expectation was also mentioned referring to the decreasing dominance of surgeons in multidisciplinary treatment decisions. Future evaluation of clinical outcomes will be facilitated by the ongoing patient-level data collection in OncoLogistic since the completeness, timeliness, and face validity of the entered data is monitored and assured by the OnkoNetwork Office.

#### Patient experience

The interviewees expected that OnkoNetwork will result in improved patient experience, since OnkoNetwork allows more time for physicians to communicate with their patients, the waiting lists are getting shorter, patients experience less need for travel and shorter waiting times on the day of diagnostics, and the number of patients intending to get enrolled outside from the catchment area of OnkoNetwork has recently increased. However, it was also noted that patients probably cannot link their fairly positive healthcare experience to the OnkoNetwork model, as fortunately they usually do not have relevant personal benchmarks in cancer care. Current monitoring of patient experience is restricted to the collection of anonymous patient satisfaction surveys that are evaluated at the department level and can’t be linked with the patients’ OnkoNetwork enrolment status.

#### Economic aspects

No economic analyses of OnkoNetwork have been conducted so far. From the perspective of the Centre, no significant economic impact is expected. The current running and operating costs of OnkoNetwork are limited to the human resources cost of about 4 full-time employees and the office leader in the OnkoNetwork Office. In general, no change is expected in the number of diagnostic visits: the number of outpatient visits per patient is expected to decrease, but this decrease is believed to be compensated for by slightly higher patient turnover. In contrast, significant economic benefits are expected to occur at the level of the national healthcare payer and the society due to a shift of the cancer stage distribution towards less advanced stages, enabling earlier and therefore less expensive treatment and better clinical outcomes.

### Financing

#### Project financing

OnkoNetwork is a regional initiative without a specific macro-level payment scheme / incentive system around it, except for a prior financial support from EU funds for the development of OncoLogistic [21]. All running and operating costs of the model are funded from regular inpatient and outpatient financing sources received from the national healthcare payer. The National Healthcare Services Center (ÁEEK), which is a governmental institute in charge of state ownership rights at the majority of hospitals in Hungary, is aware of the programme and has recently rewarded it [22]. However, no additional central funds were provided for the maintenance or development of OnkoNetwork. It is unclear whether financial incentives for the management of patient pathways will be established in the Hungarian healthcare financing system in the future.

Although the intellectual property rights of OnkoNetwork belong to the General Director of the Hospital, she considers it as a public good to improve the Hungarian healthcare system. Hence, it is available for extension to other regions subject to a financial contribution to investments in the initial and further development of the system.

#### Personal incentives

OnkoNetwork administrators of the departments and supervisor physicians typically took up the new roles on top of their existing responsibilities and tasks without significant incremental financial compensation for their additional workload. Instead, staff members reported other motivating factors, including 1) participation in an innovative and patient-oriented model, 2) working in well-designed hospital buildings with modern infrastructure, such as dedicated workstations with personal computers, 3) open communication at project meetings, 4) positive behaviour of the supervisor physicians, 5) informal social events e.g. birthday parties, and 6) the perceived improvement in individual patient pathways. Coordinators of pathways expressed that they considered the monitored patients almost as their family members and achieving optimal patient care provided personal satisfaction to themselves. Supervisor physicians were motivated by the importance of this pioneering initiative in the Hungarian healthcare setting, and by the immaterial appreciation from the colleagues and programme management. The management of OnkoNetwork acknowledges that healthcare administrators have a particularly low salary in Hungary by law, and definitely more compensation would be needed for them. In general, salaries in the Hungarian healthcare system are thought to be disappointingly low.

## Discussion

This paper provides a systematic description of planning, implementation and operation of the OnkoNetwork model programme, based on document analysis and interviews with programme initiators, managers, financial manager, involved physician and non-physician professionals, patients and their informal caregivers. OnkoNetwork was established in 2014 by the Móritz Kaposi General Hospital in Somogy county, Hungary with the aims of timely, continuous, and quality assured care of patients with first suspect of solid organ cancer in the catchment area of the participating institutions. Comorbidities frequently occur in cancer patients and need to be stabilized for optimal cancer treatment, qualifying OnkoNetwork for evaluation in the multimorbidity focused SELFIE project. OnkoNetwork has highly innovative approaches in its context (new professional roles and workflows, a supporting custom IT platform). The model is financially sustainable without receiving macro-level financial incentives for its operation at present. Its realized impacts on patient experience, health, and resource utilization is currently being investigated, with first results expected in late 2018.

Providing timely and continuous care to patients with suspected cancer need to be improved even in countries with most developed health systems. The 2015 National Cancer Program in Sweden standardized the cancer care pathways and specified time bounds for its various intervals starting from the event of “well-founded suspicion” which is defined separately for each cancer type. The objective of the Swedish initiative was to reduce waiting times, increase patient satisfaction and reduce regional inequalities in Sweden where cancer patients have generally high survival rates as compared with other EU member States [23]. In Denmark, a three-legged strategy was proposed for timely cancer diagnosis by urgent referral of patients with cancer specific symptoms and patients with unspecific but serious symptoms, and by setting up easy and fast access to “No-Yes-Clinics” or Multi-Disciplinary Diagnostic Centres for patients with common and non-serious symptoms raising a potential cancer diagnosis [24]. The UK government introduced the ‘Cancer waiting times targets’ as part of the NHS Cancer Plan in 2000, stipulating that patients with suspected cancer should be seen by a specialist within 14 days and treated within 48 days. The overall time from referral with suspected cancer to diagnosis and starting treatment should not exceed 62 days [25]. Timely cancer diagnosis in patients with unspecific symptoms is also in the forefront of research interest in the UK, where the Cancer Task Force Strategy for England considers the introduction of similar multidisciplinary centres to those established in Denmark [26]. Expecting improved stage distribution at diagnosis and patient survival by the timely diagnosis and treatment of suspected cancer patients is plausible when long delays occur in patient paths, but this assumption still calls for convincing evidence in many cancer types – sometimes with equivocal or even negative association findings due to various sources of bias, including the wait time paradox or adjustment to factors in the causal chains [27, 28]. The ongoing performance assessment of OnkoNetwork on a comprehensive range of patient health, experience, and resource utilization outcomes will meaningfully contribute to the ongoing scientific debate, providing new evidence from a former socialist EU Member State with less developed health system and with larger room for improvement in providing timely care for patients with cancer suspect.

Notably, in a recently completed EU-funded project (ICARE4EU) overviewing 101 integrated care programmes throughout the geographical Europe, only 16% of the relevant models were identified in former socialist EU Member States, and no programme could be included from the Visegrád group countries (Czech Republic, Hungary, Poland, Slovakia) and Estonia [29]. This geographic disparity in integrated care models is particularly disconcerting when differences in healthcare and social systems, economic, legal, and cultural contexts, as well as expected life years or health outcomes are considered between these regions and EU-15 Member States. Given that integrated care models are designed to fit to their surrounding macro-level environment, the transferability of experience accumulating in Western European countries to former socialist EU Member States need to be carefully assessed.

Beyond the qualitative study reported in this paper, the SELFIE project makes additional important steps to close the knowledge gap between the EU-15 and newer Member States. First, thick descriptions of three further chronic integrated care models for multimorbid patients have been approached in the CEU region, one more in Hungary [30] and two in Croatia [31, 32]. In addition, transferability assessment of 13 promising integrated care models from Western EU partner countries to CEE Member States, together with their financing/payment schemes and performance monitoring tools is specifically addressed in a dedicated work package of SELFIE. The anticipated knowledge transfer of best available European practices to less developed Member States with lower research capacity is an important opportunity in SELFIE, highlighting the significance of EU-funded research that facilitates knowledge sharing across countries and stimulates more extensive international research collaborations [2].

An inherent limitation of our study is determined by its qualitative design - the present research could not quantify the added value of OnkoNetwork to the surrounding healthcare system in Hungary. However, the conducted document analysis and stakeholder interviews allowed us to understand how OnkoNewtork was initially conceptualized and implemented, and how it could change the healthcare service provision practice in a comprehensive oncology care centre. The in-depth understanding of various stakeholder perspectives, facilitators and barriers empower the reader to learn from a carefully planned, regional initiative for timely and integrated care that turned to be implementable and sustainable in the healthcare system of a CEE Member State. Existing data on the quantitative impact of OnkoNetwork is marginal at present, due to the short duration of time since its initiation. In stakeholder interviews, an impressive list of perceived or expected impacts was gathered along the triple aim of improving *i) population health* (e.g. faster diagnosis and treatment of cancer and interfering comorbidities, improved patient compliance with diagnostics and treatment, better stage distribution at start of cancer treatment, better nutritional status of patients with head-neck tumours during therapy, and longer patient survival); *ii) patient experience* (e.g. shorter waitlists, more time for patient-physician communication, improved continuity of care); and *iii) reducing the costs or growth in costs* where significant economic benefits are expected to occur at the national healthcare payer and the society due to a shift of the cancer stage distribution towards less advanced stages. The perceived impacts promise more effective and cost-effective care for oncology patients with better physical functioning and psychological well-being. Designing a thorough quantitative study of the impact of OnkoNetwork on clinical outcomes, patient experience, and healthcare costs was guided by the findings of this qualitative study. The quantitative evaluation of OnkoNetwork is ongoing, first results are expected in late 2018.

## Conclusions

Although integrated care is in the forefront of health policy research in the EU, very few integrated care models are reported from former socialist EU Member States. Given that integrated care models are designed to fit to their specific context in the hosting country, those few models that were established in CEE countries and are sustainable represent important benchmarks for further models in this region. The comprehensive description of planning, implementation and operation of OnkoNetwork with its key success factors and barriers may support the development of new integrated care models especially in CEE countries. Findings of our study on the expected impacts of OnkoNetwork guided the planning of a subsequent quantitative study that is currently ongoing and will meaningfully contribute to the ongoing international debate on benefits associated with timeliness of cancer care.

## Additional file

Additional file 1: SELFIE Interview Protocols. Standardized protocols for the semi-structured interviews in the SELFIE project. (PDF 1666 kb)

## Abbreviations

CEE: Central and Eastern Europe
COREQ: Consolidated criteria for reporting qualitative research
ICD: International classification of disease
ICT: Information and communication technology
IT: Information technology
SELFIE: Sustainable integrated chronic care modeLs for multi-morbidity: delivery, FInancing, and performance
TB: Tumour board
