# Supplementary material for: Planning, implementation and operation of a personalized patient management system for subjects with first suspect of cancer (OnkoNetwork): system description based on a qualitative study
Source: BMC Health Serv Res. 2019 Feb 21;19:131. doi: 10.1186/s12913-019-3957-9 (PMC6383224; doi:10.1186/s12913-019-3957-9)
Supplement: Supplementary file 1 — SELFIE Interview Protocols. Standardized protocols for the semi-structured interviews in the SELFIE project. (PDF 1666 kb) [file 12913_2019_3957_MOESM1_ESM.pdf]

SELFIE

Interview Protocols

Updated Version 21st March 2016

Authors:

Thomas Czypionka

Astrid Segert

Markus Kraus

Miriam Reiss

Content:

A. Protocol for interview(s) with **manager(s) of the programme**

B. Protocol for interview(s) with **initiator(s) of the programme**

C. Protocol for interview(s) with **representative(s) of sponsor/payer organisations**

D1. Protocol for interview(s) with **physician(s)**

D2. Protocol for interview(s) with **non-physician medical staff, social staff, new professional groups**

E. Protocol for interview(s) with **informal caregiver(s)**

F. Protocol for interview(s) with **client(s) or their representative(s)**

A.

Protocol for interview(s) with
manager(s) of the programme

A. Protocol for interview(s) with <u>manager(s) of the programme</u>

Protocol for expert interviews with programme manager(s) 1/5		
**Task**	**Suggested questions – actual questions must always be adapted to suit the particular case and incorporate any prior knowledge about the interviewee**	**Remarks**
	**1.** **Brief introduction to SELFIE and the interviewer and information** about the goal of the interview (business card).	*Having a clear goal makes the interview easier.*
	**2.** Signing and exchange of the **anonymity agreement** and **declaration of consent**.	*Establishes trust and assurance.*
	**3.** Please begin by telling me which **position** you hold in the programme and **how long you have been involved with this programme**.<br><br>Follow-up questions: Professional **training and qualifications**?<br>**Why** he/she was entrusted with that particular position?	*Provides background information on the interviewee. Only ask further if this information is not provided in the interviewee's initial response. Such information assures comparability between actors.*
	**4.** Which **tasks** are you occupied with most at present?	*Provides background information on the current situation regarding programme tasks.*

For further inquiries please contact IHS-team: selfie2020@ihs.ac.at

	**5.** Please explain the **concept** behind the "XX" programme.<br><br>Follow-up questions: **Programme specifics** with regard to patients with multiple chronic conditions?<br>**Programme goals**?<br>**Target group(s)**?<br>**Institutions/people involved**?<br>**Financing scheme**?<br>**Communication concept**?<br>Which **ICT applications** used?<br>Which forms of **self-management** used?<br>Most important **effects** on the care process to date?<br>Has an **evaluation** been carried out or is one planned?	*Gather hard facts about the programme – very important when only limited written material is available, but also to identify what information on the written concept is anchored in the minds of its management.*<br><br>*If the programme focus does not lie a priori on multi-morbidity, ask about the specifics with regard to multi-morbidity for each point!*
**T2**<br>Implementation process and barriers to implementation	**6.** **How** has the "XX" programme been **implemented**?<br><br>*If interviewee has been with the programme from the start:*<br><br>What needed to be accomplished at the **start**? Which **stakeholders** were involved? What **problems** were faced at the start, and how were they handled?<br><br>*Alternative (if interviewee has not been with the programme from the start):*<br><br>What were you told about the **start phase** of the "XX" programme?<br><br>Follow-up questions: What else does it **tie in with or build on**?<br>**Facilitating factors** during implementation?<br>Was there **political commitment** and how did it manifest itself?<br>Who were **promoters of the programme**?<br>How was **change management** implemented?<br>Who **helped** with crisis situations during implementation?<br>What were **barriers/risks** and how were they handled?	*This question can be omitted if the answers were already supplied in response to Q5. If this is the case, only ask here about any points that were not directly covered – making sure in particular to inquire explicitly about the most important positive prerequisites for the development of the programme.*

For further inquiries please contact IHS-team: selfie2020@ihs.ac.at

Protocol for expert interviews with programme manager(s) 3/5		
**T3**<br>Design of delivery of care including relationships between partners involved<br><br>**T4**<br>Partnerships beyond the health care system<br><br>**T7**<br>Involvement of new professional roles	**7.** I would now like to ask you about the **work you do for the "XX" programme**. Please describe using one or more examples – and in as much detail as possible – your work as the programme manager and your experiences with the programme, in particular with regard to patients with multiple chronic conditions.<br><br>Follow-up questions: **Cooperation** with whom and in which form?<br>How are **conflicts of interest** handled?<br>Which problems in cooperation/communication with partners from **non-medical facilities**?<br>Which **new professional roles** associated with the programme?<br>**Networking** with providers of **similar programmes**?	***First main question in this interview!***<br><br>*It allows the interviewee to set his/her own points of emphasis.*<br><br>*Make sure the link to multi-morbidity is established!*
**T3**<br>Design of delivery of care including relationships between partners involved<br><br>**T6**<br>Use of self-management interventions	***8.*** *If not already covered in Q7, ask again in detail about patient-centred care:*<br><br>I would like to go into the topic of **"patient-centred care"** again in more detail. What have been your experiences with the implementation of this principle in the "XX" programme?<br><br>Follow-up questions: How is **downstream care** (along the care process) organised?<br>**Patient willingness** to get involved – and in what?<br>How are the **individual needs assessed** (is there a holistic assessment of individual?)<br>How are patients' **personal goals** taken into account?<br>What **support** is offered for **self-management**?<br>How are **informal caregivers** involved?	***Second main question in this interview!***<br><br>*This question requires a good prior analysis of the self-management interventions planned in the programme. Don't ask here about goals, but about the actual use of interventions. This also applies for Q9.*

Protocol for expert interviews with programme manager(s) 4/5		
**T5**<br>Use of ICT-applications<br><br>**T7**<br>Involvement of new professional roles	**9.** Which **<u>ICT applications</u>** do you use in the programme? What have been your **<u>experiences</u>** with the introduction and use of these **<u>applications</u>**? Can you give any specifics with regard to patients with multiple chronic conditions?<br><br><u>Follow-up questions</u>: How do these applications **<u>affect work</u>** (e.g. new tasks for professionals)?<br>What **<u>problems/barriers</u>** are there in the use of these applications? For who? (patients, professionals, informal caregivers,…)	*You can name ICT applications which you already know are used from the document analysis.*<br><br>*Establish a link to Q8, where the use of ICT in the self-management context might be mentioned (see also Document 5 for a list of ICTs).*<br>*Don't ask about goals, but about what is really done.*
**TS**<br>Financing and payment schemes	***10.*** *<u>If not addressed in Question 7, ask explicitly again about the following:</u>*<br>What were your **<u>experiences</u>** with the **<u>financing procedures</u>** in the "XX" programme? What works well? Where do you as programme manager see problems?<br><br>What experiences have you had with the **<u>payment system</u>** in the "XX" programme?<br><br><u>Follow-up questions</u>: **<u>Regular financing/payment</u>** or **<u>special arrangements</u>**?<br>Cooperation with **<u>financing organisation(s)</u>**?<br>What **<u>problems</u>** are there with the financing scheme?<br>**<u>Bonus systems</u>** for **<u>professionals</u>** – what kind?<br>**<u>Bonus system/nudges</u>** for **<u>insurees/patients</u>** – what kind?	*Aims at giving a first impression about financing/payment system, that is explored in more detail in a separate interview for WP3*
**T8**<br>Evaluation<br><br>**T7**<br>Involvement of new professional roles	**11.** In your view, what are the most important **<u>effects</u>** of the "XX" programme, in particular with regard to patients with multiple chronic conditions?<br><br><u>Follow-up questions</u>: Effects on **<u>work processes</u>**?<br>Effects on **<u>communication</u>**?<br>Effects on **<u>costs</u>**?<br>Which **<u>new professional roles</u>**?<br>Effects on **<u>health parameters</u>** of the patients?<br>Effects on patient **<u>quality of life</u>**?	*Address positive and negative aspects equally.*

For further inquiries please contact IHS-team: selfie2020@ihs.ac.at

**T8**<br>Evaluation	**12.** *If not answered for Q6:*<br>How do you **document** and assess the results of your efforts in the "XX" programme?<br><br>Follow-up questions: What are **focal points** of documentation?<br>Documentation **intervals**?<br>**Internal evaluations** – what has been evaluated?<br>**External evaluations** – what has been evaluated?<br>What does the **monitoring** and **quality assurance system** look like?	
	**13.** If you were to summarise your own experiences, what makes the **"XX" programme innovative** in comparison to regular care or other integrated care programmes?<br><br>Follow-up questions: **Characteristic features** of integrated care for patients with multiple chronic conditions?<br>Biggest **challenge** for the future?<br>**Ideas** for the future?	*Opportunity to sum up at the end of the interview.*
	**Thank you for talking to me today!**<br>**Would you be interested in receiving the results of the research project?**<br>**If yes → make a note of the interviewee's e-mail address (if not already on file).**	

B.

Protocol for interview(s) with
initiator(s) of the programme

B. Protocol for interview(s) with <u>initiator(s) of the programme</u>

Protocol for expert interviews with programme initiator(s) 1/3		
**Task**	**Suggested questions – actual questions must always be adapted to suit the particular case and incorporate any prior knowledge about the interviewee**	**Remarks**
	**1.** **Brief introduction to SELFIE and the interviewer and information** about the goal of the interview (business card).	*Identical for all expert interviews.*
	**2.** Signing and exchange of the **anonymity agreement** and **declaration of consent**.	*Identical for all expert interviews.*
	**3.** Could you start by telling me something about yourself? What is your **vocational background** and your **professional position**?	*Background information on the interviewee.*

Protocol for expert interviews with programme initiator(s) 2/3		
**T2**<br>Implementation process and barriers to implementation<br><br>**T3**<br>Design of delivery of care including relationships between partners involved<br><br>**T7**<br>Involvement of new professional roles	**4.** I am particularly interested in how the **"XX" programme** came to be. You have been involved in the programme from the start / since … and were one of its initiators. Can you tell me in detail how and why the **idea** was born and how you helped to bring it to life?<br><br>Follow-up questions: What was the **basic idea**?<br>What did the idea **tie in with** or **build on**?<br>What were **expectations** with regard to care for patients with multiple chronic conditions?<br>What did the implementation **procedure** look like?<br>How was **contact** established?<br>Who were **co-initiators/helpers**?<br>**Facilitating factors** during implementation?<br>**Barriers/risks** and how were they handled?<br>**Conflicts** and how were they resolved?<br>**New professional roles** – part of the basic idea or originated as a result of the work?<br>What were important **legal and political circumstances** during the implementation of the programme?	***Main question in this interview!***<br><br>*Let the interviewee talk until he/she is finished, even if this takes a long time.*<br><br>*Ask about multi-morbidity aspects!*<br><br>*Along the question about barriers/risks/facilitators keep in mind the macro-level of the framework (legal/political etc. framework)*
**T4**<br>Partnerships beyond the health care system	***5.*** *If not already addressed in response to Q4:*<br><br>I have another question regarding the preparations for the practical implementation of the programme. How did you in particular **establish contact** with representatives of **non-medical facilities**? Were any special activities necessary to integrate them?<br><br>Follow-up questions: What are **advantages** of such partnerships?<br>What are/were **barriers** (e.g. different professional jargon, other institutional practices) – how are they handled?	*Depends on the answers to Q4.*

For further inquiries please contact IHS-team: selfie2020@ihs.ac.at

Protocol for expert interviews with programme initiator(s) 3/3

**T3**<br>Design of delivery of care including relationships between partners<br><br>**T6**<br>Use of self-management interventions<br><br>**TS**<br>Financing and payment schemes	***6.*** *If the interviewee currently has an active role in the programme:*<br><br>I would now like to ask you about the **work** you are currently doing in the "XX" programme. Please describe your task(s) in and experiences with the programme, in particular with regard to patients with multiple chronic conditions.<br><br>Follow-up questions: Concrete **tasks** and timeframe?<br>How are **joint decisions** taken?<br>**Different ways of handling** patient groups?<br>Experiences with **self-management**?<br>**Cooperation** with whom and in which form?<br>Resolution of **conflicts of interest** with partners?<br>**Networking** with providers of **similar programmes**?<br>Experiences with **financing and payment scheme**?	*Omit this question if interviewee does not have an active role in the programme.*
**T8**<br>Evaluation	**7.** What do you think are the most important **effects** of the "XX" programme and how are these effects **evaluated**?<br><br>Follow-up questions: Effects on **communication**?<br>Effects on **costs**?<br>Effects on **health outcomes** of the patients?<br>Effects on patients' **quality of life**?<br>Effects on **satisfaction** of involved professionals?	*Avoid getting people into a defensive position when asking about evaluation.*
	**8.** If you were to summarise your experiences with the programme in two sentences, what would be the key **successes** and what are the most important **problems** that need to be resolved in integrated care for patients with multiple chronic conditions?<br><br>Follow-up questions: **Ideas** for the future?	*Opportunity to sum up at the end of the interview.*
**Thank you for talking to me today!**<br>**Would you be interested in receiving the results of the research project?**<br>**If yes → make a note of the interviewee's e-mail address (if not already on file).**		

For further inquiries please contact IHS-team: selfie2020@ihs.ac.at

C.

Protocol for interview(s) with **representative(s) of sponsor/payer organisations**

C. Protocol for interview(s) with <u>representative(s) of sponsor/payer organisations</u>

Protocol for expert interviews with representative(s) of sponsor/payer organisations 1/4		
**Task**	**Suggested questions – actual questions must always be adapted to suit the particular case and incorporate any prior knowledge about the interviewee**	**Remarks**
	**1.** **Brief introduction to SELFIE and the interviewer and information** about the goal of the interview (business card).	*Identical for all expert interviews.*
	**2.** Signing and exchange of the **anonymity agreement** and **declaration of consent**.	*Identical for all expert interviews*
	**3.** Could you start by telling me something about yourself? What is your **vocational background** and your **professional position**?	*Background information on the interviewee.*
	**4.** What role does the “XX” programme play in your work?	*Background information on the relevance of the work on this programme for the interviewee.*

For further inquiries please contact IHS-team: selfie2020@ihs.ac.at

Protocol for expert interviews with representative(s) of sponsor/payer organisations 2/4		
**TS**<br>Financing and payment schemes	**5.** Could you please briefly describe the **<u>financing and payment system</u>** for the "XX" programme! We are particularly interested here in where funding comes from, how payments are made and how funds are distributed in the programme.<br><br><u>Follow-up question</u>: What was the **<u>idea behind this</u>**?<br>What can you say about the **<u>effects</u>** of this system?	*Provides information on existing concepts/intentions and experiences.*
**T2**<br>Implementation process and barriers to implementation	**6.** I would now like to ask you about the financing decision for the "XX" programme. Were you involved in this decision?<br><br>*<u>If interviewee has been involved with the programme from the start:</u>*<br><br>What were the underlying **<u>strategic considerations</u>** behind the financing decision for the programme? What **<u>expectations</u>** did you have of the programme with respect to its advantages for patients with multiple chronic conditions? What expectations did you have with regard to how costs and benefits of the programme would develop?<br><br>*<u>Alternative (if interviewee has not been involved with the programme from the start):</u>*<br><br>What have you been told about the **<u>strategic goals</u>** and expectations for the "XX" programme? By **<u>expectations</u>**, we mean here the expectations regarding the advantages for patients with multiple chronic conditions and with regard to how the costs and benefits of the programme would develop.<br><br><u>Follow-up questions</u>: **<u>Main intention</u>** in funding the programme?<br>Most important aspects of the programme for the provision of **<u>improved care</u>**?<br>Funding through **<u>existing resources</u>** or **<u>newly created/provided resources</u>**?<br>Financing system introduced following **<u>internal discussions</u>** or the use of **<u>external expertise</u>**?<br>What **<u>problems</u>** were faced in drawing up the financing concept?	***First main question in this interview!***<br><br>*Important here is whether the financing organisation also initiated the programme or whether resources were provided in response to a project initiative (e.g. from doctors).*

For further inquiries please contact IHS-team: selfie2020@ihs.ac.at

Protocol for expert interviews with representative(s) of sponsor/payer organisations 3/4		
**TS**<br>Financing and payment schemes	**7.** I would ask you now to describe your **<u>experiences with the financing and payment system</u>** of the “XX” programme in as much detail as possible. How do the financial incentives work (if applied)? What problems have you been confronted with? Where did you make changes and why?<br><br><u>Follow-up questions</u>: What **<u>incentives</u>** are there **<u>to participate in the programme</u>** for different stakeholders? **<u>Bonus system/nudges</u>** for **<u>patients</u>**?<br>**<u>Compatibility</u>** with **<u>regular prices/payment tariffs</u>**?<br>**<u>Biggest problems</u>** – and how are they handled?<br>Ex-post **<u>adaptations to financing and payment</u>** – if yes, which and why?<br>**<u>Conflicts of interest</u>** – and how were they resolved?	***Second main question in this interview!***
**T4**<br>Partnerships beyond the health care system	***8.*** *<u>If not already addressed in response to Q7</u>:*<br>The “XX” programme also involves providers outside the health sector. How does the **<u>financing/payment system</u>** work for **<u>non-medical partner institutions</u>**?<br><br><u>Follow-up questions</u>: How is the cooperation **<u>organised</u>**?<br>What were/are **<u>problems</u>** in this regard – and how are they handled?	*Omit this question if there are no corresponding relationships.*

<table>
<tr><td colspan="3">Protocol for expert interviews with representative(s) of sponsor/payer organisations 4/4</td></tr>
<tr><td>T8
Evaluation</td><td>9. When you compare your expectations for the “XX” programme (which you described at the start of this interview) to the actual experience with the programme, where have these <u>expectations been met</u> and where have they <u>not been met</u>? Which <u>evaluation criteria</u> do you use to come to this assessment?

<u>Follow-up questions</u>: Effects on <u>health outcomes</u> of patients?
Effects on <u>costs</u> (cost savings – where/additional costs – where)?
Experiences when defining the <u>evaluation categories</u>?
Experiences with <u>evaluation reports</u>?
<u>Satisfaction</u> with the evaluation system?</td><td>Focus here on the actual evaluation work; if the interviewee is not involved in the evaluation process, then focus instead on the effects.</td></tr>
<tr><td>TS
Financing and payment schemes

T8
Evaluation</td><td>10. If you were to sum up your experiences with the “XX” programme, how would you assess its financing and payment system? Is this concept <u>fit for the future</u> and is it <u>suitable for general use</u> for the integrated care for patients with multiple chronic conditions?

<u>Follow-up questions</u>: <u>Sustainability</u> of financing and payment system?
Most important <u>effects</u> of the system?
<u>Greatest challenge</u> for the future?
<u>Ideas</u> for the future?</td><td>Opportunity to sum up at the end of the interview.</td></tr>
<tr><td colspan="3">Thank you for talking to me today!
Would you be interested in receiving the results of the research project?
If yes → make a note of the interviewee’s e-mail address (if not already on file).</td></tr>
</table>

For further inquiries please contact IHS-team: selfie2020@ihs.ac.at

D1.

Protocol for interview(s) with **physician(s)**

D1. Protocol for interview(s) with <u>physician(s)</u>

Protocol for expert interviews with physician(s) 1/5		
**Task**	**Suggested questions – actual questions must always be adapted to suit the particular case and incorporate any prior knowledge about the interviewee**	**Remarks**
	**1.** **Brief introduction to SELFIE and the interviewer and information** about the goal of the interview (business card).	*Identical for all expert interviews.*
	**2.** Signing and exchange of the **anonymity agreement** and **declaration of consent**.	*Identical for all expert interviews.*
	**3.** Could you start by telling me something about yourself? What **qualifications** do you have and **how long** have you been working as a physician at XX?	*Background information on the interviewee.*
	**4.** Could you please briefly tell me what **work** you are predominantly occupied with at present – independent of the focus of our interview.<br>*Alternative question:*<br>Which problems currently occupy most of your time in your work?	*Background information on the relevance of the programme work for the interviewee.*

For further inquiries please contact IHS-team: selfie2020@ihs.ac.at

Protocol for expert interviews with physician(s) 2/5		
**T3** Design of delivery of care including relationships between partners involved<br><br>T4 Partnerships beyond the health care system	**5.** I would now like to ask you about your **work in the "XX" programme**. Could you give me one or more examples to describe the work you do for patients with multiple chronic conditions in particular in the "XX" programme?<br><br>Follow-up questions: **How long** have you been involved with the "XX" programme?<br>Is participation **autonomous** or **by instruction**?<br>How are **diagnoses and treatment decisions made**?<br>How are **individual needs assessed** – is there a holistic assessment of the patient?<br>How are the patient's **personal goals** taken into account?<br>How is **risk stratification** in the programme done?<br>Are **individual care plans** used and what do they look like?<br>How is the **shared decision making** approach applied?<br>What does communication/cooperation with providers of **non-medical services** look like?<br>How are **conflicts** with programme partners resolved?<br>**Effects on workload** – additional workload (where, e.g. documentation)/reduced workload (where)?<br>Specific advantages/problems for **patients with multiple chronic conditions**?<br>How are issues of **polypharmacy** and **guideline interaction** dealt with?<br>**Networking** with partners in other programmes?<br>How are **informal caregivers** involved in the programme?	***Main question in this interview!***<br><br>*This question allows the interviewee to set his/her own priorities – the responses will be very different in each case.*<br><br>*Make sure to consider the interviewee's professional position when formulating any follow-up questions.*

**T6**<br>Use of self-management interventions	***6.*** <u>*If not covered adequately in response to Q5:*</u><br>I would now like to ask about the <u>**doctor-patient relationship**</u> in more detail. What has changed through the programme in this respect?<br><br><u>Follow-up questions</u>: Problems for patients in <u>**gaining access to the programme**</u> – how are they handled?<br>How willing are patients to <u>**assume personal responsibility**</u> – and where?<br>How is <u>**patient empowerment**</u> achieved?<br>What <u>**gaps in patient knowledge/problems in communicating**</u> are there?<br>What are <u>**main barriers**</u> in doctor-patient relationship?	*Don't ask about goals or objectives but about what is actually done.*
**T5**<br>Use of ICT-applications	***7.*** <u>*If not covered adequately in response to Q5, ask again specifically:*</u><br>Which ICT applications do you use in the "XX" programme? What have been your <u>**experiences**</u> with these applications?<br><br><u>Follow-up questions</u>: Influence on <u>**workload**</u>?<br>What <u>**problems**</u> are there in the use of such applications?	*Ask about all ICTs mentioned in the programme documentation.*
**T7**<br>Involvement of new professional roles	***8.*** <u>*If not covered adequately in response to Q5, ask again specifically:*</u><br>Are there <u>**education or training measures**</u> in person-centred care provided that you have participated in? If yes, what have been your <u>**experiences**</u> with such measures? If not, how do you think such measures could be <u>**helpful**</u>?	*Informs about specific training requirements associated with person-centred care.*

**TS**<br>Financing and payment schemes	***9.*** *<u>If not covered adequately in response to Q5, ask again specifically:</u>*<br><br>Do you feel that the work you do in the “XX” programme is <u>**appreciated**</u> and <u>**adequately paid**</u>? What have been your <u>**experiences**</u> in this regard?<br><br><u>Follow-up questions</u>: <u>**Satisfied/not satisfied**</u> and with what?<br>What would be your <u>**wishes**</u> in this regard?	*Can be omitted if not appropriate.*
**T8**<br>Evaluation<br><br>**T7**<br>Involvement of new professional roles	**10.** What do you personally consider to have been the most important <u>**effects**</u> of your work in the “XX” programme?<br><br><u>Follow-up questions</u>: More <u>**time**</u> for patients?<br>Effects on <u>**relationship to patients**</u>?<br>Effects on efficiency of <u>**work processes**</u>?<br><u>**Changes**</u> to your own <u>**working style**</u>?<br>Changes in the <u>**distribution of tasks**</u> among all of the involved personnel?<br>Effects on <u>**communication**</u>?<br>Effects on <u>**costs**</u>?<br>Effects on <u>**health outcomes**</u> of the participating patients?<br>Effects on patient <u>**quality of life**</u>?	*Important here are not the effects indicated in the evaluation report, but those which the interviewee personally considers to be important.*
**T8**<br>Evaluation	***11.*** *If not covered in response to Q10:*<br><br>How do you <u>**document**</u> the results of your efforts in the “XX” programme and how are they <u>**assessed**</u>?<br><br><u>Follow-up questions</u>: What are <u>**focal points**</u> of documentation?<br>Documentation <u>**intervals**</u>?<br><u>**Internal evaluation**</u> – what has been evaluated?<br><u>**External evaluation**</u> – what has been evaluated?<br><u>**Monitoring/quality assurance system**</u> – what does it look like?<br><u>**Experiences**</u> with being monitored/evaluated?	*The focus here is on the efforts of the interviewee himself/herself. However, he/she can also include his/her own thoughts on the documentation efforts of other partners.*

Protocol for expert interviews with physician(s) 5/5		
**T3** Design of delivery of care including relationships between partners involved	**12.** If you were to sum up your experiences with your work in the programme, what would you consider to be the **most important successes** of this work in comparison to other programmes and regular care?<br><br>Follow-up questions: **Specifics** of integrated care for patients with multiple chronic conditions?<br>Greatest **challenge** for the future?<br>**Ideas** for the future?	*Opportunity to sum up at the end of the interview*
**Thank you for talking to me today!**<br>**Would you be interested in receiving the results of the research project?**<br>**If yes → make a note of the interviewee's e-mail address (if not already on file).**		

For further inquiries please contact IHS-team: selfie2020@ihs.ac.at

D2.

Protocol for interview(s) with
non-physician medical staff, social staff, new professional groups

D2. Protocol for interview(s) with <u>non-physician medical staff, social staff, new professional groups</u>

Protocol for expert interviews with non-physician medical staff, social staff, new professional groups 1/4		
**Task**	**Suggested questions – actual questions must always be adapted to suit the particular case and incorporate any prior knowledge about the interviewee**	**Remarks**
	**1.** **<u>Brief introduction to SELFIE and the interviewer and information</u>** about the goal of the interview (business card).	*Identical for all expert interviews.*
	**2.** Signing and exchange of the **<u>anonymity agreement</u>** and **<u>declaration of consent</u>**.	*Identical for all expert interviews.*
	**3.** Could you start by telling me something about yourself? What **<u>qualifications</u>** do you have and **<u>how long</u>** have you been working at YY?	*Background information on the interviewee.*
	**4.** I would now like to ask you about your work **<u>in the "XX" programme</u>**. How long have you been involved in the programme?	*Background information on the relevance of the programme work for the interviewee.*

For further inquiries please contact IHS-team: selfie2020@ihs.ac.at

**T3**<br>Design of delivery of care including relationships between partners involved<br><br>**T4**<br>Partnerships beyond the health care system	**5.** Did you receive any special **training** for your work with patients with multiple chronic conditions? What format did/does this training take?<br><br>Follow-up questions: How did this influence your **confidence in your work**?<br>Who is your **designated contact person** for any questions?<br>Specifics for professionals from **non-medical facilities**?<br>If no training, how do you think that special training/education measures could be **helpful**?	*For interviewees from non-medical facilities, ask specifically about the relationship to medical facilities.*
**T3**<br>Design of delivery of care including relationships between partners involved<br><br>**T4**<br>Partnerships beyond the health care system<br><br>**T7**<br>Involvement of new professional roles	**6.** I would now like to gain a picture of your work. Can you please tell me **what you did at work yesterday** and what role caring for patients with multiple chronic conditions in particular played in your day? Who were you in contact with in this regard? What format does the care provided to these patients take?<br><br>*Alternative question:*<br><br>Please tell me about a typical working day in the "XX" programme. What **do you do** for patients with multiple chronic conditions in particular? Who do you normally communicate with in doing so and what questions do you have? What problems do you face in your work? Please take your time with your answer. I won't interrupt you. If I have any questions, I'll ask them when you've finished talking.<br><br>Follow-up questions: What kind of **patient groups** are cared for?<br>**Cooperation** primarily with **whom** and **how**?<br>Involved in **joint diagnoses and treatment decisions** – how?<br>If in a hospital: your involvement in the **discharge process** – how?<br>**Changes** to your **working style** and **work processes** through the programme – what changed?	***First main question in this interview!***<br><br>*This question allows the interviewee to set his/her own priorities – the responses will be very different in each case*<br><br>*Make sure to consider the interviewee's position when formulating any follow-up questions, e.g. working in a hospital, in a GP practice or a care facility.*

For further inquiries please contact IHS-team: selfie2020@ihs.ac.at

Protocol for expert interviews with non-physician medical staff, social staff, new professional groups 3/4		
**T6**<br>Use of self-management interventions<br><br>**T5**<br>Use of ICT-applications	**7.** The programme requires the **<u>active participation of the patients</u>**. Can you tell me about your experiences in this regard? How do the patients react? How do they deal with uncertainties and questions?<br><br><u>Follow-up questions</u>: **<u>Example</u>** of successful self-management?<br>How are **<u>individual needs assessed</u>** – is there a holistic assessment of the patient?<br>Use of **<u>ICT applications</u>** – what is used and what are experiences with these applications?<br>How are patients' **<u>personal goals</u>** taken into account?<br>What effects on **<u>health outcomes</u>** of patients?<br>What effects on **<u>patient quality of life</u>**?<br>What effects on **<u>patient satisfaction</u>**?<br>How **<u>willing</u>** are patients to assume responsibility?<br>What **<u>barriers</u>** for patients to get access to the programme and how are they handled?<br>What **<u>communication problems</u>** are there and how are they handled?<br>What **<u>other barriers</u>** are there?<br>How are **<u>informal caregivers</u>** involved in the programme?	***Second main question in this interview!***
**T8**<br>Evaluation	**8.** How do you yourself **<u>document</u>** the results of the integrated care? Do you **<u>share data</u>** with other cooperating facilities – if yes, how do you share data and which data do you share?<br><br><u>Follow-up questions</u>: What are the **<u>focal points</u>** of documentation?<br>Which **<u>software</u>** do you use and what are experiences with this?<br>How are successes **<u>measured and assessed</u>** – is there a monitoring/quality assurance system and what does it look like?	*If necessary, have the interviewee show you the corresponding documentation.*

Protocol for expert interviews with non-physician medical staff, social staff, new professional groups 4/4		
**T3**<br>Design of delivery of care including relationships between partners involved<br><br>**T4**<br>Partnerships beyond the health care system	**9.** I would now like to gain a picture of how **communication** among everyone involved in the programme works. When and in which way are experiences, successes and any barriers encountered in the programme **discussed**? **How** are you involved in this process?<br><br>Follow-up questions: What **barriers** are there to open exchange about successes and problems?<br>What **problems** are there in **communication/cooperation** with other professions (e.g. physicians, non-medical professions) are there – how are they handled?<br>In what way are such discussions **helpful**?<br>Cooperation with **partners from other facilities** – how does that happen, what format does it have?	*Can also include informal get-togethers as well as official meetings.*
**TS**<br>Financing and payment schemes	**10.** Do you feel that the work you do in the "XX" programme is **appreciated** and **adequately paid**? What have been your **experiences** in this regard?<br><br>Follow-up questions: **Satisfied/not satisfied** and with what?<br>What would be your **wishes** in this regard?	*Can be omitted if not appropriate.*
**T3**<br>Design of delivery of care including relationships between partners involved	**11.** Finally, I would like to ask you to **sum up**. What do you think are the **main benefits** of integrated care specifically for patients with multiple chronic conditions and their caregivers? What are the greatest **barriers** to the provision of such care?<br><br>Follow-up questions: **Ideas** for the future or things you would like to see **improved**?	*Opportunity to sum up at the end of the interview*
**Thank you for talking to me today!**<br>**Would you be interested in receiving the results of the research project?**<br>**If yes → make a note of the interviewee's e-mail address (if not already on file).**		

For further inquiries please contact IHS-team: selfie2020@ihs.ac.at

E.

Protocol for interview(s) with **informal caregiver(s)**

E. Protocol for interview(s) with **informal caregiver(s)**

Protocol for expert interviews with informal caregiver(s) 1/4		
**Task**	**Suggested questions – actual questions must always be adapted to suit the particular case and incorporate any prior knowledge about the interviewee**	**Remarks**
	**1.** **Brief introduction to SELFIE and the interviewer and information** about the goal of the interview (business card).	*Identical for all expert interviews.*
	**2.** Signing and exchange of the **anonymity agreement** and **declaration of consent**.	*Identical for all expert interviews.*
	**3.** You care for Ms./Mr. AA. I would like to talk to you about that in a moment. But first, could you tell me something **about yourself**? How old are you? What do/did you work as? What is your relationship to Ms./Mr. AA?	*Background information on the interviewee.*

For further inquiries please contact IHS-team: selfie2020@ihs.ac.at

Protocol for expert interviews with informal caregiver(s) 2/4		
**T3**<br>Design of delivery of care including relationships between partners involved<br><br>**T4**<br>Partnerships beyond the health care system	**4.** Thank you. I would now like to move on to your **care work**.<br>The first thing I'd like to know is: **How long** you have been caring for Ms./Mr. AA? But I am also particularly interested in what you **do** and the care you provide. Could you please describe that for me in as much detail as possible? Which care work do you provide? Please tell precisely what you do, when you do it, who you **coordinate** this work with, what problems you encounter, who **helps** you in an emergency, etc. Anything and everything you think is important.<br>I won't interrupt you, but I may have some follow-up questions when you are finished.<br><br>Follow-up questions: What are the **main problems** you encounter in your care work?<br>In which way do you **cooperate** with **(non-)medical care partners** – what are your experiences in this regard?	***First main question in this interview!***<br><br>*Responses could also cover Q6 – this is fine, allow it to happen.*
**T3**<br>Design of delivery of care including relationships between partners involved<br><br>**T6**<br>Use of self-management interventions	**5.** I am particularly interested in how you are involved in the "XX" programme. Please try to recall: What was it like at the **start**? How did you become involved with the programme? **Who** contacted you and in which way? What **happened next** and what has happened **since**?<br><br>Follow-up questions: **How long** involved in the programme?<br>**Expectations** of participation in the programme?<br>**Training** in self-management – how and by whom?<br>What **incentives** were/are provided by the programme?<br>**Patient's view** of participation in the programme?	***Second main question in this interview!***

**T3**<br>Design of delivery of care including relationships between partners involved<br><br>**T4**<br>Partnerships beyond the health care system	***6.*** *If not covered in response to Q4:*<br>If you were to compare the care you provided previously with that provided now in the programme, what has **changed**? What do you do differently now and what has **changed for the patient**, Ms./Mr. AA?<br><br>Follow-up questions: What has **improved** for you, e.g. work-life balance, confidence in care work, social security?<br>What are your **fears/uncertainties**?<br>Who are your **main contact persons**?<br>How are you involved in **development of care plan** and **treatment decisions**?<br>**Your satisfaction** with the informal care work and its results?<br>**Patient's satisfaction**?<br>Adequate **recognition** for the work you do? E.g. special **payment/reward** for the informal care work provided?	*This question allows the interviewee to set his/her own priorities – the responses will be very different in each case*<br><br>*When formulating your questions, remember that you are not talking to a medical professional.*
**T5**<br>Use of ICT-applications	***7.*** *If not covered in response to Q6:*<br>What about the **technical/computer** aspects? Do you use any technical equipment for your care work? What things do you now have to do in this regard that you didn't need to before joining the programme?<br><br>Follow-up questions: **Training** for ICT application – what did it look like, how helpful was it?<br>Does it make care **easier** or is it an **additional burden**?<br>How well are ICT applications **accepted** by the patient?<br>Where do you get professional **help** in case of problems?	*If necessary, have the interviewee show you.*<br><br>*The question can be omitted if no ICT applications are used.*

For further inquiries please contact IHS-team: selfie2020@ihs.ac.at

**T3**<br>Design of delivery of care including relationships between partners involved<br><br>**T5**<br>Use of ICT-applications<br><br>**T6**<br>Use of self-management interventions	**8.** Finally, I would like to ask you for **your opinion** on the following: Was **participating in the "XX" programme** the right decision? **Why** was this decision right or not right? Please tell me what you think are the most important reasons.<br><br>Follow-up questions: **Ideas** for the future or things you would like to see **improved**? **Ideas** to improve use of **ICT applications** and **self-management interventions**?	*Opportunity to sum up at the end of the interview*

Thank you for talking to me today!
Would you be interested in receiving the results of the research project?
If yes → make a note of the interviewee's e-mail address (if not already on file).

F.

Protocol for interview(s) with **client(s) or their representative(s)**

F. Protocol for interview(s) with <u>client(s) or their representative(s)</u>

Protocol for interviews with client(s) or their representative(s) 1/3		
**Task**	**Suggested questions – actual questions must always be adapted to suit the particular case and incorporate any prior knowledge about the interviewee**	**Remarks**
	**1.** **Brief introduction to SELFIE and the interviewer and information** about the goal of the interview.	*Don't forget to mention the person who set up the interview.*
	**2.** Signing and exchange of the **anonymity agreement** and **declaration of consent**.	*Make sure you clearly explain that no names will be included in the analysis report.*
	**3.** Ms./Mr. AA, How are you **feeling today**?<br><br>Follow-up questions: Which **chronic illnesses?**<br>Which other **health problems** (pain etc.)?	*Background information on the interviewee's multi-morbidity.*
	**4.** I would like to ask you first to tell me something **about yourself**. How old are you? Are you married or do you live with a partner? Do you have any children? What work do/did you do? Things like that!	*Background information on the interviewee.*

For further inquiries please contact IHS-team: selfie2020@ihs.ac.at

Protocol for interviews with client(s) or their representative(s) 2/3		
	**5.** You are taking part in the "XX" programme, and I would now like to talk to you in detail about that. Can you tell me **<u>how you initially became involved</u>** with the programme? Did your doctor talk to you about it or how did it come about? Please, go ahead and tell me what you can remember.<br><br><u>Follow-up questions</u>: **<u>How long</u>** have you participated in programme?<br>What were your **<u>information sources</u>**?<br>What were your **<u>expectations</u>** of participation?<br>What were your **<u>fears/reservations</u>**?	*Encourage the interviewee to talk.*<br><br>*Use informal language and expressions.*
**T3**<br>Design of delivery of care including relationships between partners involved<br><br>**T4**<br>Partnerships beyond the health care system<br><br>**T7**<br>Involvement of new professional roles	**6.** I would now like to find out about the **<u>care</u>** you receive. Could you tell me – in as much detail as possible – what kind of care you are receiving? **<u>Who</u>** is treating you and/or caring for you? Which **<u>facilities</u>** do you go to? How often do you go there? What do you do there? Which things are done in your **<u>home</u>**?<br>Please take plenty of time and tell me what treatment and care you receive from AA and BB! I won't interrupt, but I may have some follow-up questions when you are finished.<br><br><u>Follow-up questions</u>: What **<u>changed</u>** in comparison to treatment/care before joining programme?<br>Who are main **<u>contact persons</u>**?<br>Contacts with **<u>non-medical professionals</u>** - who?<br>How does communication work? What **<u>communication problems</u>** are there?	***First main question in this interview!***<br><br>*Encourage the interviewee to tell his/her story.*<br><br>*This question allows the interviewee to set his/her own priorities – the responses will be very different in each case.*
**T3**<br>Design of delivery of care including relationships between partners involved	***7.*** *<u>If not covered in response to Q6</u>:*<br><br>Do you have a **<u>say in your own care plan</u>**? Who do you talk to about that? How does this happen?<br><br><u>Follow-up questions</u>: How are your **<u>personal goals, preferences and priorities</u>** taken into account?<br>What **<u>uncertainties</u>** do you have – how are they dealt with?<br>Who are your **<u>main contact persons</u>**?<br>Are you **<u>satisfied</u>** with level of participation?	*If the interviewee has reservations about talking about this, encourage him/her to do so by asking questions; if necessary, remind the interviewee that everything he/she says will be treated anonymously.*

For further inquiries please contact IHS-team: selfie2020@ihs.ac.at

**T5**<br>Use of ICT applications<br><br>**T6**<br>Use of self-management interventions	**8.** I would like to ask again more precisely about your **own role in your treatment**. Have you yourself taken on new tasks, e.g. XX? Please describe to me – in as much detail as possible –what you need to (be able to) do and how you cope!<br><br>Follow-up questions: **Uncertainties in self-management** – where and how are they handled?<br>**Help with problems** – who helps and how?<br>Are you **satisfied** with level of participation?<br>Use of **ICT applications** – experiences and problems?<br>**Training** for use of ICT applications – sufficient?	***Second main question in this interview!***<br><br>*If necessary, have the interviewee show you.*
	**9.** Finally, I would like to ask you for **your opinion** on the following: If you think back to the last few months/years: Was the decision to **participate** in the "XX" programme the right one? **Why** was this decision right or not right? Please tell me what you think are the most important reasons.<br><br>Follow-up questions: **Worries/problems** with respect to your care and the programme?<br>**Ideas** for the future or things you would like to see **improved**?<br>**Ideas** to improve use of **ICT applications** and **self-management interventions**?	*Opportunity to sum up at the end of the interview*
**Thank you for talking to me today.**<br>**Would you be interested in receiving the results of the research project?**<br>**If yes → make a note of the interviewee's e-mail address (if not already on file).**		

For further inquiries please contact IHS-team: selfie2020@ihs.ac.at
